# Hybrid IRBM-BPNN Approach for Error Parameter Estimation of SINS on Aircraft

**DOI:** 10.3390/s19173682

**Published:** 2019-08-24

**Authors:** Weilin Guo, Yong Xian, Daqiao Zhang, Bing Li, Leliang Ren

**Affiliations:** Xi’an Research Institute of High Technology, Xi’an 710025, China

**Keywords:** integrated navigation, information fusion, error parameter estimation, hybrid approach, restricted Boltzmann machine, BP neural network, pulse signal

## Abstract

To realize the error parameter estimation of strap-down inertial navigation system (SINS) and improve the navigation accuracy for aircraft, a hybrid improved restricted Boltzmann machine BP neural network (IRBM-BPNN) approach, which combines restricted Boltzmann machine (RBM) and BP neural network (BPNN), is proposed to forecast the inertial measurement unit (IMU) instrument errors and initial alignment errors of SINS. Firstly, the error generation mechanism of SINS is analyzed, and initial alignment error model and IMU instrument error model are established. Secondly, an unsupervised RBM method is introduced to initialize BPNN to improve the forecast performance of the neural network. The RBM-BPNN model is constructed through the information fusion of SINS/GPS/CNS integrated navigation system by using the sum of position deviation, the sum of velocity deviation and the sum of attitude deviation as the inputs and by using the error parameters of SINS as the outputs. The RBM-BPNN structure is improved to enhance its forecast accuracy, and the pulse signal is increased as the input of the neural network. Finally, we conduct simulation experiments to forecast and compensate the error parameters of the proposed IRBM-BPNN method. Simulation results show that the artificial neural network method is feasible and effective in forecasting SINS error parameters, and the forecast accuracy of SINS error parameters can be effectively improved by combining RBM and BPNN methods and improving the neural network structure. The proposed IRBM-BPNN method has the optimal forecast accuracy of SINS error parameters and navigation accuracy of aircraft compared with the radial basis function neural network method and BPNN method.

## 1. Introduction

Strap-down inertial navigation system (SINS) has the advantages of high reliability, continuous outputs, independence and strong anti-interference capability and has been widely used in the field of aircraft navigation. However, the gyroscopes and accelerometers of inertial measurement unit (IMU) inevitably exhibit drift and bias caused by manufacturing process and other reasons. Measurement errors accumulate with time, thereby affecting navigation accuracy. IMU instrument errors seriously affect the stability and accuracy of aircraft flights, especially for high-speed, long-range and long-endurance ones, such as ballistic missiles, hypersonic vehicles.

An aircraft requires initial alignment before launching to determine the attitude matrix, and the initial alignment also has a huge impact on the navigation accuracy of the aircraft. In practice, initial alignment errors are inevitable and should be considered. Overall, the error parameters of SINS, including IMU instrument errors and initial alignment errors, should be compensated and corrected to improve the navigation accuracy and environmental adaptability of aircraft. At present, the estimation and correction methods of SINS error parameters include filtering algorithms, intelligent optimization algorithms and artificial neural network methods.

Filtering algorithms including Kalman filtering (KF) and its variants are widely used in the field of integrated navigation. Zhang et al. [1] proposed an estimation method for spacecraft attitude and position based on cubature KF (CKF), and the simulation results showed that the proposed method had higher attitude and position estimation accuracy than extended KF (EKF) algorithm. Zhao et al. [2] used an extended $H_{\infty }$ filtering algorithm to realize the information fusion of INS, and the simulation results showed that the method could improve the navigation accuracy of INS for aerospace vehicles. Ding et al. [3] proposed an adaptive KF algorithm, that used an adaptive process noise scaling method based on covariance to improve the filter performance, and the simulation results showed that the method was highly reliable and robust for the error estimation of navigation parameters of GPS/INS integrated navigation system. However, filtering algorithms have complicated derivation of state equation and long filtering time, and KF algorithms are required to determine the precise priori knowledge of system models and noise characteristics.

The error propagation of SINS is a complex nonlinear system problem. Intelligent optimization algorithms have certain advantages in solving the error estimation problem of SINS because they are not constrained by search space limitations; does not require to have assumptions, such as continuous and derivative existence; and possess hidden parallelism, huge search space, simple structure and easy to realize. Zhu et al. [4] proposed a particle swarm optimization (PSO) algorithm to solve the parameter selection problem of compass alignment circuit of SINS, and the experimental results showed that the proposed PSO algorithm was effective and feasible for compass alignment of SINS. Guo et al. [5] used an online identification method of initial alignment errors based on adaptive PSO to realize the high-precision initial alignment of SINS for ballistic missile, and the simulation results showed that the adaptive PSO had a fast convergence velocity and high convergence accuracy, that can effectively improve the navigation accuracy of SINS. He et al. [6] employed a genetic algorithm to optimize the compass alignment parameters for SINS, and the experimental results showed that the method could effectively adapt to the compass alignment problem under the large misalignment angle. The above studies have used intelligent optimization algorithms to the optimize the initial alignment errors of INS, without considering the IMU instrument error estimation of SINS, and the initial alignment accuracy should be further improved. Few studies have been conducted on the parameter optimization of IMU instrument errors using intelligent optimization algorithms. Dai et al. [7] proposed an accelerometer error calibration method based on PSO algorithm to reduce the dependence of IMU calibration on high-precision turntable, and the method could achieve rapid accelerometer error calibration. However, the proposed method belonged to static calibration based on turntable and only calibrated the accelerometer error parameters. Of course, intelligent optimization algorithms have disadvantages of huge amount of calculation and relatively poor real-time performance because of the need for the calculation of fitness of each population.

Artificial neural network methods have good real-time calculation and strong fitting ability, without identifying the internal structure of the system. With the continuous development of artificial intelligence technology, artificial neural network methods have played an increasingly important role in the field of inertial navigation [8]. Hasan and Saadeddin et al. [9,10] adopted the adaptive neuro-fuzzy inference system (ANFIS) based on current and previous INS data to forecast the INS errors when GPS signals are interrupted, and the simulation results showed that ANFIS had a good estimation effect. Ning et al. [11] used radial basis function neural network (RBFNN) and enhanced adaptive robust KF method for GNSS/INS integrated navigation system in complex urban suburbs, and field tests showed that the method could improve the position navigation accuracy when the GNSS signal was interrupted. Yao et al. [12] applied an improved multi-layer perceptual network (MLP) to estimate the pseudo GPS position for improving the performance of GPS/INS integrated navigation system during GPS interruption, and the method had better forecast performance than traditional artificial neural network.

However, the network initial values of the above neural network forecast methods are randomly generated, and the neural network training effect is sensitive to the network initial values. A neural network easily falls into local optimal when the selected initial values are not properly determined. In addition, the forecast effect of a single network model is limited. Scholars at home and abroad have combined and improved different models to further improve the navigation accuracy of SINS and overcome the shortcomings of a single neural network model. Wang et al. [13] proposed a method to optimize the structure of BP neural network (BPNN) based on PSO, and used the optimized weight and threshold for the initialization of the neural network. The INS position error of surface ship was forecasted by using BP neural network model when the star sensor of INS/CNS integrated navigation system did not work properly. Fan et al. [14] constructed a hybrid algorithm based on genetic algorithm and BP neural network to estimate the navigation state parameter errors of GPS/DRS simple integrated navigation system. The disadvantages of local optimal and slow convergence speed of traditional algorithms were overcome, and the method had higher precision and stability compared with conventional KF algorithms. Sharaf et al. [15] used wavelet transformation to process the data noise of INS/GPS integrated navigation system and utilized RBFNN to forecast the INS position errors. 

With the improvement of neural network, the above research methods have made the network initial values more reasonable and improved the forecast effect of error parameters and navigation accuracy of INS. However, in the navigation field, few studies have been conducted on the optimization of initial weight and threshold of neural network through unsupervised machine learning. The forecast performance of network models can be improved when the initial values of neural network are initialized through machine learning.

Boltzmann machine (BM) is a type of symmetrically connected and random neural network model with no self-feedback [16]. The output of BM neuron is only 0 or 1, where 1 indicates the active state and 0 indicates the inactive state. The state value can be obtained through probability statistics [17]. The BM model has powerful unsupervised learning ability to learn and extract complex information from data. However, due to the existence of full connections between each neuron within the visible and hidden layer, BM also has obvious disadvantages, such as complex structure, long network training time and low efficiency. The expected sample distribution is difficult to obtain. On this basis, Smolensky [18] proposed a Restricted Boltzmann machine (RBM) model to solve the problem on the basis of the BM model.

RBM is a bidirectional probability graph model based on statistical laws. RBM has unsupervised learning ability, easy reasoning, parallelism and fault tolerance because of its excellent characteristics and special structure. RBM is one of the basic structural units that constitute a deep neural network model, and multiple RBMs stacking can be constructed to form a deep belief neural network (DBN) [19]. RBM has good forecast effect and strong feature information extraction capability, and has been successfully utilized to solve regression, classification, image feature extraction, forecast and other problems [20,21,22,23].

A hybrid improved restricted Boltzmann machine BP neural network (IRBM-BPNN) approach combining RBM and BPNN is proposed to forecast and estimate the IMU instrument errors and initial alignment errors of SINS based on the above research and analyses and relevant research achievements. Firstly, the overall scheme of SINS error parameter estimation is designed, and IMU instrument error model and initial alignment error model are established. Then, the RBM-BPNN forecast model is constructed based on SINS/GPS/CNS integrated navigation system measurement information by using the sum of position deviation, the sum of velocity deviation and the sum of attitude deviation as the network inputs and SINS error parameters as the network outputs. The model firstly uses an unsupervised learning method to train the RBM, assigns the weight and bias of the trained RBM to the BPNN for completing the network initialization, and uses a BP algorithm to conduct supervised training on the entire neural network for improving the network forecast performance. The structure of RBM-BPNN is improved to enhance the forecast accuracy of SINS error parameters. The pulse signal is increased as the input of neural network on the basis of the original navigation deviation, and SINS error parameters are forecasted and estimated by the IRBM-BPNN. Finally, the reliability and effectiveness of IRBM-BPNN are verified through simulation experiments. The main innovative contributions of this paper are summarized as follows:

(1) Traditional neural network methods for integrated navigation system have focused on the estimation and compensation of navigation state parameter error, where the estimation of IMU instrument errors has been rarely reported. What’s more, few studies have been conducted on parameter estimation and compensation of SINS that consider the IMU instrument errors and initial alignment errors. In this study, an artificial neural network method is used to simultaneously estimate and forecast the IMU instrument errors and initial alignment errors, and the research content is in-depth and challenging;
(2) For the design of neural network structure, previous studies have mainly regarded the navigation state parameters or navigation deviations of integrated navigation system as the inputs or outputs of the neural network and have not utilized the pulse information from IMU. In this paper, the network structure is improved, the gyroscope pulse signal and accelerometer pulse signal are taken as the inputs of the neural network, and the data are more fully utilized, which are conducive to the estimation of SINS error parameters;
(3) In this paper, an RBM model is used to initialize the BPNN for obtaining good initial network values to avoid the neural network falling into local optimal. An RBM-BPNN forecast model is constructed by combining RBM and BPNN networks to improve the forecast accuracy of SINS error parameters.
(4) Unsupervised feature learning mechanism is introduced in the field of integrated navigation and SINS error parameters estimation, which enhances the processing and adaptability of the neural network model to the measurement data of integrated navigation system and provides new insight into the error parameter estimation and navigation accuracy improvement of SINS.

The rest structure of this paper is organized as follows. In Section 2, the overall scheme design of SINS error parameter estimation is introduced. The initial alignment error model and IMU instrument error model of SINS are established in Section 3. Section 4 constructs the RBM-BPNN forecast model. In Section 5, the structure of RBM-BPNN is improved, and simulation and comparison experiments are provided to verify the feasibility of the neural network method. Section 6 summarizes the conclusions, and expounds the future research direction of SINS error estimation.

## 2. Overall Error Estimation Scheme Design

**Figure 1:**
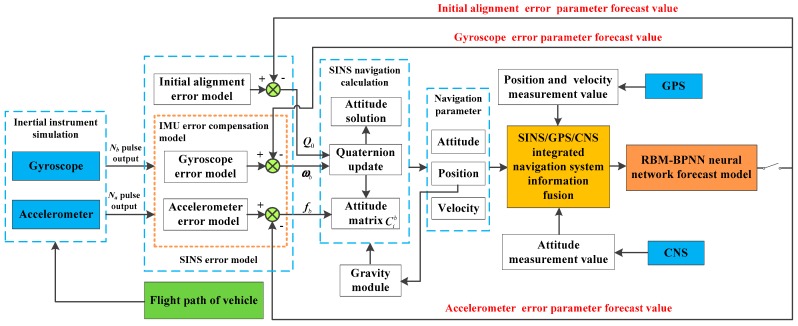
Overall error estimation scheme of RBM-BPNN method.

At present, multiple source information fusion is an important means to improve navigation accuracy. The overall error estimation scheme of the RBM-BPNN method is designed based on SINS/GPS/CNS integrated navigation system information to effectively estimate and identify SINS error parameters of aircraft, as shown in Figure 1. Where $N_{a}$ and $N_{b}$ represent the accelerometer pulse output and the gyroscope pulse output, respectively [24]; $f_{b}$ and $\omega_{b}$ represent the specific force and the angle velocity after error compensation, respectively; $Q_{0}$ represents the initial value of quaternion; $C_{i}^{b}$ represents the attitude matrix between the inertial coordinate and body coordinate.

As shown in Figure 1, the movement of an aircraft generates signal excitation to the IMU, in which the gyroscope and accelerometer output pulse signal $N_{b}$ and $N_{a}$, respectively. The pulse signals are then input into the SINS error model. The SINS error model includes the initial alignment error model, gyroscope error model and accelerometer error model. The specific force $f_{b}$ and angular velocity $\omega_{b}$ are calculated and output by the IMU error compensation model. The initial value of quaternion $Q_{0}$ is output by the initial alignment error model. SINS navigation calculation module is conducted to calculate the SINS navigation state parameters, including attitude, position and velocity, based on the above output information. In addition, auxiliary navigation system GPS can measure the position and velocity values of the aircraft, and CNS can measure the attitude values of the aircraft.

Then, multiple source information is fused based on the SINS/GPS/CNS integrated navigation system measurements. The specific fusion strategy in this paper can be described as follows. The sum of position deviation and the sum of velocity deviation between SINS and GPS are calculated based on the position output and velocity output of SINS/GPS integrated navigation system. The sum of attitude deviation between SINS and CNS is calculated based on the attitude output of the SINS/CNS integrated navigation system.

Finally, the sum of position deviation, the sum of velocity deviation and the sum of attitude deviation after information fusion are input into the RBM-BPNN forecast model, and then the initial alignment errors and IMU instrument errors of SINS are forecasted and estimated by the RBM-BPNN method. Once the estimation of error parameters is complete, the switch in Figure 1 is disconnected. Then the initial alignment error parameter forecast values are fed back to the initial alignment error model to update the initial value of quaternion $Q_{0}$; the IMU instrument error parameter forecast values are fed back to the IMU error compensation model to realize the error compensation and correction. The estimation of SINS error parameters is finally completed to meet the navigation accuracy requirements of aircraft.

## 3. SINS Error Model Establishment

According to the principle of SINS measurement, there are two main sources of navigation error.

The first source is the initial measurement reference error of SINS. The calculation of navigation state parameters of SINS is conducted under the given reference conditions, where the attitude matrix established by SINS will have an error when the given reference has an error, which will cause navigation error in measurement and navigation calculation of SINS. Therefore, the effect of initial alignment errors must be considered. 

The second source is the measurement model error of SINS. Theoretically, the measurement error of SINS should be small and the navigation accuracy of aircraft should be high when the IMU instrument error coefficient calibrated at the ground stage is accurate after the real-time error compensation by the IMU error compensation model. However, because the bias and drift of IMU are variable in different start each time, the calibration values of the IMU instrument error coefficient are not applicable. The measurement error of SINS accumulates over time, which affects the navigation accuracy of aircraft. Therefore, the effect of IMU instrument errors must also be considered.

### 3.1. Initial Alignment Error Model of SINS

Ideally, a vertically launched aircraft has no initial attitude errors before take-off. However, initial alignment errors, including initial pitch angle error $\Delta \varphi_{0}$, initial yaw angle error $\psi_{0}$ and initial roll angle error $\gamma_{0}$, exist during the actual launch because of installation error, aiming error and other effects. The initial alignment errors affect the establishment of the attitude matrix $C_{i}^{b}$, and $C_{i}^{b}$ can be described by quaternion, which is shown as follows:

(1) $$C_{i}^{b}=\left[\begin{array}{ccc} q_{1}^{2}+q_{0}^{2}-q_{2}^{2}-q_{3}^{2} & 2(q_{1}q_{2}-q_{0}q_{3}) & 2(q_{0}q_{2}+q_{1}q_{3})\\ 2(q_{1}q_{2}+q_{0}q_{3}) & q_{0}^{2}+q_{2}^{2}-q_{1}^{2}-q_{3}^{2} & 2(q_{2}q_{3}-q_{0}q_{1})\\ 2(q_{1}q_{3}-q_{0}q_{2}) & 2(q_{0}q_{1}+q_{2}q_{3}) & q_{0}^{2}+q_{3}^{2}-q_{1}^{2}-q_{2}^{2} \end{array}\right]$$

where $q_{0}$, $q_{1}$, $q_{2}$ and $q_{3}$ represent the quaternion, and the initial value of quaternion $Q_{0}=(q_{0},q_{1},q_{2},q_{3})$ is calculated as:

(2) $$\{\begin{array}{c} q_{0}=q_{00}-\frac{\gamma_{0}}{2}q_{20}\\ q_{1}=q_{10}+\frac{\gamma_{0}}{2}q_{30}\\ q_{2}=q_{20}+\frac{\gamma_{0}}{2}q_{00}\\ q_{3}=q_{30}-\frac{\gamma_{0}}{2}q_{10} \end{array}$$

where

(3) $$\{\begin{array}{l} q_{00}=\frac{\sqrt{2}}{2}(1-\frac{\Delta \varphi_{0}}{2})\\ q_{10}=-\frac{\psi_{0}}{2}q_{00}\\ q_{20}=\frac{\psi_{0}}{2}q_{30}\\ q_{30}=\frac{\sqrt{2}}{2}(1+\frac{\Delta \varphi_{0}}{2}) \end{array}$$

As shown in Equations (1)–(3), it can be seen that the initial alignment errors determine the initial value of quaternion $Q_{0}$, and the initial value of quaternion determines the calculation result of the attitude matrix $C_{i}^{b}$. Therefore, as long as the initial alignment error parameter values are estimated accurately, the attitude matrix can be corrected by the above initial alignment error model, thus the navigation accuracy of aircraft can be improved.

### 3.2. IMU Instrument Error Model of SINS

As the core component of SINS, IMU is mainly composed of gyroscope and accelerometer. The drift term of gyroscope error coefficient mainly includes the zero-order error coefficient and first-order error coefficient, and the installation error coefficient of gyroscope remains unchanged after the IMU is manufactured and stored in a stable period. The zero-order error coefficient and the first-order error coefficient of accelerometer will change, except that the installation error coefficient of accelerometer basically remains same. Therefore, the error model of the accelerometer and gyroscope can be obtained as follows [25].

The accelerometer error model can be formulated as:

(4) $$\{\begin{array}{c} \delta f_{bx}=\Delta K_{0x}+\Delta K_{1x}f_{bx}+\varepsilon_{ax}\\ \delta f_{by}=\Delta K_{0y}+\Delta K_{1y}f_{by}+\varepsilon_{ay}\\ \delta f_{bz}=\Delta K_{0z}+\Delta K_{1z}f_{bz}+\varepsilon_{az} \end{array}$$

where $\delta f_{bx}$,$\delta f_{by}$ and $\delta f_{bz}$ represent the apparent acceleration errors of aircraft in body coordinate; $\Delta K_{0x}$, $\Delta K_{0y}$ and $\Delta K_{0z}$ represent the zero-order coefficient errors of accelerometer; $\Delta K_{1x}$, $\Delta K_{1y}$ and $\Delta K_{1z}$ represent the first-order coefficient errors of accelerometer; $f_{bx}$, $f_{by}$ and $f_{bz}$ represent the component of the specific forces in body coordinate; $\varepsilon_{ax}$, $\varepsilon_{ay}$ and $\varepsilon_{az}$ represent the measurement white noises of accelerometer.

The gyroscope error model can be formulated as:

(5) $$\{\begin{array}{c} \delta \omega_{bx}=\Delta D_{0x}+\Delta D_{1x}f_{bx}+\varepsilon_{gx}\\ \delta \omega_{by}=\Delta D_{0y}+\Delta D_{1y}f_{bx}+\varepsilon_{gy}\\ \delta \omega_{bz}=\Delta D_{0z}+\Delta D_{1z}f_{bx}+\varepsilon_{gz} \end{array}$$

where $\delta \omega_{bx}$, $\delta \omega_{by}$ and $\delta \omega_{bz}$ represent the angle velocity errors of aircraft in body coordinate; $\Delta D_{0x}$, $\Delta D_{0y}$ and $\Delta D_{0z}$ represent the zero-order drift coefficient errors of gyroscope; $\Delta D_{1x}$, $\Delta D_{1y}$ and $\Delta D_{1z}$ represent the first-order coefficient errors of gyroscope; $\varepsilon_{gx}$, $\varepsilon_{gy}$ and $\varepsilon_{gz}$ represent the measurement white noises of gyroscope.

In addition, the establishment and description of the IMU error compensation model and the SINS navigation calculation model can be referred in [5], which will not be repeated here. The navigation state parameters of SINS, such as attitude, position and velocity information, can be calculated based on the IMU error compensation model and SINS navigation calculation model.

## 4. RBM-BPNN Method Design for SINS Error Estimation

### 4.1. Introduction of Basic Principles of RBM

**Figure 2:**
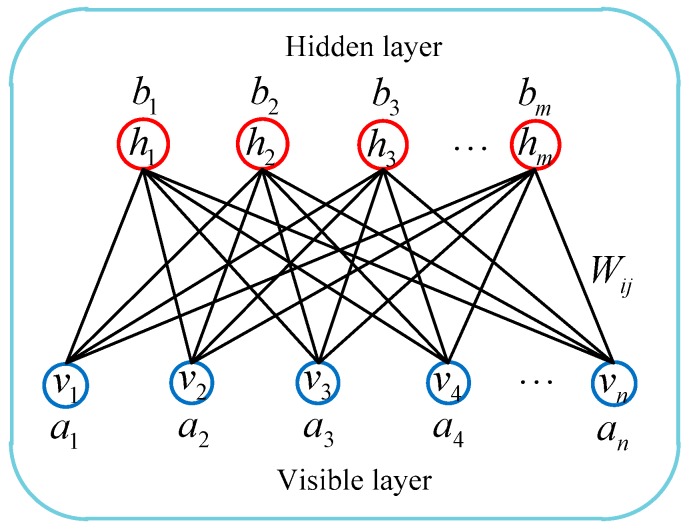
RBM network structure.

As an undirected graph model, RBM is a special topology from BM based on energy functions. An RBM consists of a visible layer and a hidden layer. The visible layer represents the input data, and the hidden layer represents the feature information extracted from the input data. Unlike the BM model, there is no connection between the neurons within the same layers for the RBM model. Consequently, RBM has the advantages of simple structure and easy learning. The RBM network structure is shown in Figure 2.

Where n represents the number of neurons in the visible layer, and $v=\left\{v_{1},v_{2},\cdots ,v_{n}\right\}$ represents the state of the visible units; m represents the number of neurons in the hidden layer, and $h=\left\{h_{1},h_{2},\cdots ,h_{m}\right\}$ represents the state of the hidden units; W represents the connection weight between the visible layer and the hidden layer; $a=\left\{a_{1},a_{2},\cdots ,a_{n}\right\}$ and $b=\left\{b_{1},b_{2},\cdots ,b_{m}\right\}$ represent the biases of the visible layer and the hidden layer, respectively. For a given state (v,h), the energy function of RBM network model is defined as follows:

(6) $$E(v,h)=-a^{T}v-b^{T}h-h^{T}Wv$$

When RBM network parameter θ=(a,b,W) is determined, joint probability distribution P(v,h) can be obtained based on the energy function given by Equation (6), that is:

(7) $$\left\{ \begin{array}{l} P(v,h)=\frac{1}{Z}e^{-E(v,h)}\\ Z=\sum_{v,h}e^{-E(v,h)} \end{array}\right.$$

where Z represents the normalization factor, also known as the partition function. For practical engineering problems, visible layer v represents the actual measurement data, and its edge probability distribution P(v) can be calculated based on joint probability distribution P(v,h), that is:

(8) $$P(v)=\sum_{h}P(v,h)=\frac{1}{Z}\sum_{h}e^{-E(v,h)}$$

The conditional probability distributions of visible layer and hidden layer neurons should be determined to estimate parameter θ of the RBM model. On the basis of related theory of conditional probability distribution and reference [16], when the visible layer is known, condition probability distribution $P(h_{j}=1|v)$ of hidden layer neuron $h_{j}=1$ can be expressed as:

(9) $$P(h_{j}=1|v)=\frac{1}{1+e^{-(b_{j}+\sum_{i}v_{i}W_{ij})}}=f(b_{j}+\sum_{i}v_{i}W_{ij})$$

Similarly, when the hidden layer is known, condition probability distribution $P(v_{i}=1|h)$ of visible layer neuron $v_{i}=1$ can be expressed as:

(10) $$P(v_{i}=1|h)=\frac{1}{1+e^{-(a_{i}+\sum_{j}W_{ij}h_{j})}}=f(a_{i}+\sum_{j}W_{ij}h_{j})$$

As shown in Equation (9) and (10), $f(x)=\frac{1}{1+e^{-x}}$ is the sigmoid activation function. 

To determine the RBM model, parameter θ=(a,b,W) should be calculated to ensure that the probability distribution represented by RBM is consistent with the sample data distribution. The edge probability distribution P(v) is shown in Equation (8), and its log-likelihood function l(θ) can be described as:

(11) l(θ)=log(P(v))

By maximizing the log-likelihood function l(θ), we can get the parameter estimation $\theta^{*}$ of RBM model, that is:

(12) $$\theta^{*}=\underset{\theta }{\arg\;\max}l(\theta )=\underset{\theta }{\arg\;\max}\log(P(v))=\underset{\theta }{\arg\;\max}\log(\frac{1}{Z}\sum_{h}e^{-E(v,h)})$$

The partial derivative of the log-likelihood function l(θ) is taken with respect to parameters (a,b,W), and we obtain:

(13) $$\left\{ \begin{array}{l} \frac{\partial l(\theta )}{\partial a_{i}}={\langle v_{i}\rangle }_{data}-{\langle v_{i}\rangle }_{\mathrm{mod}el}\\ \frac{\partial l(\theta )}{\partial b_{j}}={\langle h_{j}\rangle }_{data}-{\langle h_{j}\rangle }_{\mathrm{mod}el}\\ \frac{\partial l(\theta )}{\partial W_{ij}}={\langle v_{i}h_{j}\rangle }_{data}-{\langle v_{i}h_{j}\rangle }_{\mathrm{mod}el} \end{array}\right.$$

where ${\langle \cdot \rangle }_{P}$ represents the mathematical expectation of the distribution P, and ‘data’ and ‘model’ represent the probability distribution P(h|v) and P(v,h) respectively. From Equation (13) we can see that the calculation of the partial derivative needs to obtain a joint probability distribution P(v,h), and it can be seen from Equation (7) that the acquisition of P(v,h) requires to calculated the normalization factor Z, while the calculation complexity of Z value is large. Aimed at the problem, Gibbs sampling is usually used to obtain an approximation of the probability distribution P(v,h), but Gibbs sampling requires a large number of sampling steps, which results in low training efficiency of RBM.

To overcome this difficulty, Hintion proposed a contrast divergence (CD) algorithm to effectively solve the problem of RBM learning efficiency [26]. The expectations in Equation (13) are approximately estimated by using the CD algorithm, and the calculation results of the partial derivative are expressed as follows:

(14) $$\left\{ \begin{array}{l} \Delta a_{i}={\langle v_{i}\rangle }_{data}-{\langle v_{i}\rangle }_{recon}\\ \Delta b_{j}={\langle h_{j}\rangle }_{data}-{\langle h_{j}\rangle }_{recon}\\ \Delta W_{ij}={\langle v_{i}h_{j}\rangle }_{data}-{\langle v_{i}h_{j}\rangle }_{recon} \end{array}\right.$$

where $\Delta a_{i}$, $\Delta b_{j}$ and $\Delta W_{ij}$ are the approximate values of partial derivatives $\frac{\partial l(\theta )}{\partial a_{i}}$, $\frac{\partial l(\theta )}{\partial b_{j}}$ and $\frac{\partial l(\theta )}{\partial W_{ij}}$, respectively, and ‘recon’ represents the reconstructed RBM model distribution. Finally, the estimated values of parameters $\left(a_{i},b_{j},W_{ij}\right)$ of the RBM model can be obtained as follows.

(15) $$\left\{ \begin{array}{l} a_{i}=a_{i}+\eta \Delta a_{i}\\ b_{j}=b_{j}+\eta \Delta b_{j}\\ W_{ij}=W_{ij}+\eta \Delta W_{ij} \end{array}\right.$$

where η represents learning rate of RBM model.

### 4.2. RBM-BPNN Forecast Model Construction

RBM and BPNN theories state that an output layer is added to the two-layer RBM network, and RBM is expanded into a three-layer NN, which is the RBM-BPNN model.

**Figure 3:**
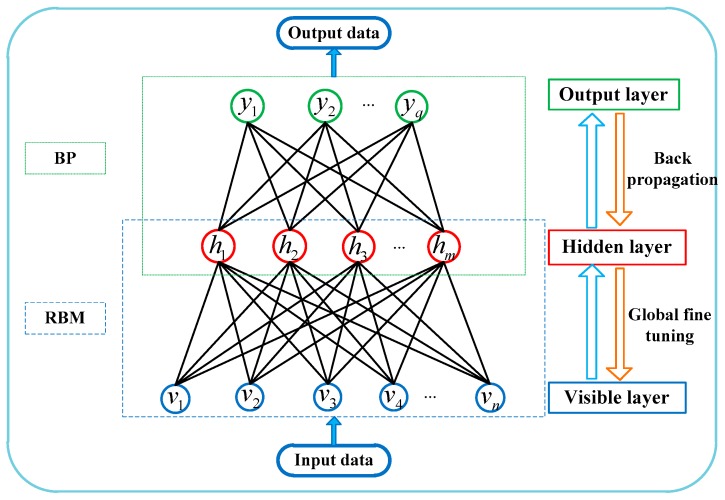
RBM-BPNN network structure.

As shown in Figure 3, the input and output data of the neural network should be determined before determining the RBM-BPNN structure. As previously mentioned in Section 2, the inputs of RBM-BPNN structure can be obtained through the fusion of multiple information sources of the SINS/GPS/CNS integrated navigation system, that is, the sum of position deviation, the sum of velocity deviation and the sum of attitude deviation.

The sum of position deviation and the sum of velocity deviation can be calculated based on the position and velocity output values of the SINS/GPS integrated navigation system as follows:

(16) $$\left\{ \begin{array}{l} \delta x=\sum_{t=0}^{T_{k}}\left(x_{SINS}^{}(t)-x_{GPS}^{}(t)\right)\\ \delta y=\sum_{t=0}^{T_{k}}\left(y_{SINS}^{}(t)-y_{GPS}^{}(t)\right)\\ \delta z=\sum_{t=0}^{T_{k}}\left(z_{SINS}^{}(t)-z_{GPS}^{}(t)\right) \end{array}\right.$$

where δx, δy and δz are the sum of position deviations between SINS and GPS from time 0 to time $T_{k}$; $x_{GPS}(t)$, $y_{GPS}(t)$ and $z_{GPS}(t)$ are the position parameters measured by GPS at t moment; $x_{SINS}(t)$, $y_{SINS}(t)$ and $z_{SINS}(t)$ are the position parameters outputted by SINS at t moment. $T_{k}$ is the flight time of aircraft; time step t is 0.1 s.

The sum of velocity deviations is calculated as follows:

(17) $$\left\{ \begin{array}{l} \delta v_{x}=\sum_{t=0}^{T_{k}}\left(v_{x}{}_{SINS}^{}(t)-v_{x}{}_{GPS}^{}(t)\right)\\ \delta v_{y}=\sum_{t=0}^{T_{k}}\left(v_{y}{}_{SINS}^{}(t)-v_{y}{}_{GPS}^{}(t)\right)\\ \delta v_{z}=\sum_{t=0}^{T_{k}}\left(v_{z}{}_{SINS}^{}(t)-v_{z}{}_{GPS}^{}(t)\right) \end{array}\right.$$

where $\delta v_{x}$, $\delta v_{y}$ and $\delta v_{z}$ are the sum of velocity deviations between SINS and GPS from time 0 to time $T_{k}$; $v_{x}{}_{GPS}(t)$, $v_{y}{}_{GPS}(t)$ and $v_{zGPS}(t)$ are the velocity parameters measured by GPS at t moment; $v_{x}{}_{INS}^{}(t)$, $v_{y}{}_{SINS}(t)$ and $v_{z}{}_{SINS}(t)$ are the velocity parameters outputted by SINS at t moment.

The sum of attitude deviation can be calculated based on the attitude output value of the SINS/CNS integrated navigation system as follows:

(18) $$\left\{ \begin{array}{l} \delta \varphi =\sum_{t=0}^{T_{k}}\left(\varphi_{SINS}^{}(t)-\varphi_{CNS}^{}(t)\right)\\ \delta \psi =\sum_{t=0}^{T_{k}}\left(\psi_{SINS}^{}(t)-\psi_{CNS}^{}(t)\right)\\ \delta \gamma =\sum_{t=0}^{T_{k}}\left(\gamma_{SINS}^{}(t)-\gamma_{CNS}^{}(t)\right) \end{array}\right.$$

where δφ, δψ and δγ are the sum of attitude deviations between SINS and CNS from time 0 to time $T_{k}$; $\varphi_{CNS}^{}(t)$, $\psi_{CNS}^{}(t)$ and $\gamma_{CNS}^{}(t)$ are the attitude parameters measured by CNS at t moment; $\varphi_{SINS}^{}(t)$, $\psi_{SINS}^{}(t)$ and $\gamma_{SINS}^{}(t)$ are the attitude parameters outputted by SINS at t moment.

As shown in Equation (1) to (5), the initial alignment errors $\left(\Delta \varphi_{0},\psi_{0},\gamma_{0}\right)$ and gyroscope instrument errors $\left(\Delta D_{0x},\Delta D_{0y},\Delta D_{0z},\Delta D_{1x},\Delta D_{1y},\Delta D_{1z}\right)$ and accelerometer instrument errors $\left(\Delta K_{0x},\Delta K_{0y},\Delta K_{0z},\Delta K_{1x},\Delta K_{1y},\Delta K_{1z}\right)$ are the error parameters that need to be forecasted and estimated in this paper, and also the outputs of RBM-BPNN forecast model. Therefore, RBM-BPNN model is a nine-input and fifteen-output neural network structure, the mapping relationship between the inputs and outputs can be expressed as follows:

(19) $$\begin{array}{l} \left(\Delta \varphi_{0},\psi_{0},\gamma_{0},\Delta D_{0x},\Delta D_{0y},\Delta D_{0z},\Delta D_{1x},\Delta D_{1y},\Delta D_{1z},\Delta K_{0x},\Delta K_{0y},\Delta K_{0z},\Delta K_{1x},\Delta K_{1y},\Delta K_{1z}\right)\\ =f(\delta x,\delta y,\delta z,\delta v_{x},\delta v_{y},\delta v_{z},\delta \varphi ,\delta \psi ,\delta \gamma ) \end{array}$$

The mapping relationship of Equation (19) through analytic calculation is difficult to obtain because the error propagation of SINS is a complex nonlinear process, and the resolution between IMU instrument errors and navigation state parameter deviation is difficult to derive. The RBM-BPNN method has powerful mapping ability for nonlinear systems, which does not require to master and derive the error propagation within SINS, and can directly fit the physical model based on input and output data. The RBM-BPNN structure can be determined by training the sample data, and the mapping relationship between the inputs and outputs is directly established, which is beneficial to solving the problem in this paper. 

The specific flow of the error parameters forecast of SINS based on the RBM-BPNN method is described as follows:

(1) Data preprocess. The sample data are collected based on SINS/GPS/CNS integrated navigation system measurement and SINS error parameter information. Then, the sample data are normalized and mapped to the interval of [0, 1], which is expressed as:
  (20) $$\tilde{x^{\prime }}=(\tilde{x}-{\tilde{x}}_{\min})/({\tilde{x}}_{\max}-{\tilde{x}}_{\min})$$
  where ${\tilde{x}}_{\max}$ and ${\tilde{x}}_{\min}$ are the maximum value and minimum value of the sample data, respectively;
(2) The training sample is input into the RBM model, the RBM structure is trained by using a greedy learning algorithm without supervision, and the deep feature information of the input data is extracted. Then, the weight and bias of the RBM structure are determined;
(3) The BPNN is initialized based on the RBM training results. The weight and threshold between input layer and hidden layer of BPNN are replaced by the weight and bias of RBM;
(4) The weight and threshold value of the entire network of RBM-BPNN are fine-tuned by using a BP algorithm, and the mapping relationship between the input layer and output layer is obtained, that is, the RBM-BPNN structure;
(5) The trained RBM-BPNN model is evaluated by testing samples, and the generalization ability and error parameter forecast effect of the model are verified.

We adopt two criteria to evaluate the forecast performance of SINS error parameters based on the RBM-BPNN model, including mean absolute percentage error (MAPE) and mean absolute error (MAE).

The calculation equations of MAPE and MAE are shown in Equation (21) and (22), respectively.

(21) $$MAPE=\frac{1}{n_{s}}\sum_{i=1}^{n_{s}}R_{i}=\frac{1}{n_{s}}\sum_{i=1}^{n_{s}}\left|\frac{x_{i}-{\tilde{x}}_{i}^{}}{{\tilde{x}}_{i}^{}}\right|\times 100\%$$

(22) $$MAE=\frac{1}{n_{s}}\sum_{i=1}^{n_{s}}\left|x_{i}-{\tilde{x}}_{i}\right|$$

where $n_{s}$ represents the total number of samples; $x_{i}$ represents the error parameter forecast value of *i*th sample; ${\tilde{x}}_{i}^{}$ represents the error parameter actual value of *i*th sample; $R_{i}$ represents the relative error of the error parameter of *i*th sample. It is noted that the relative error is set to 100% when the relative error of the error parameter is more than 100%. The smaller the MAPE and MAE values are, the higher the estimation accuracy, and the better the performance of the RBM-BPNN model will be.

## 5. Simulation Experiments and Result Analysis

### 5.1. Simulation Condition Settings

The simulation conditions of SINS/GPS/CNS integrated navigation system and initial alignment error parameters are set as follows:

(1) GPS and CNS navigation errors. Position measurement error and altitude measurement error of GPS are 10.0 m, velocity measurement error of GPS is 0.1 m/s, and attitude measurement error of CNS is 10″ [2,27,28];
(2) The initial launch parameters of aircraft. The coordinate of the launch point is (118°E, 32°N, 0.0 m), and the launch azimuth is 90° [28];
(3) The initial alignment error parameters of SINS. The standard deviation of the initial pitch angle error is 60″, the standard deviation of the initial yaw angle error is 60″, and the standard deviation of the initial roll angle error is 90″ [29];
(4) IMU instrument error parameter settings are shown in Table 1 [24].

**Figure 4:**
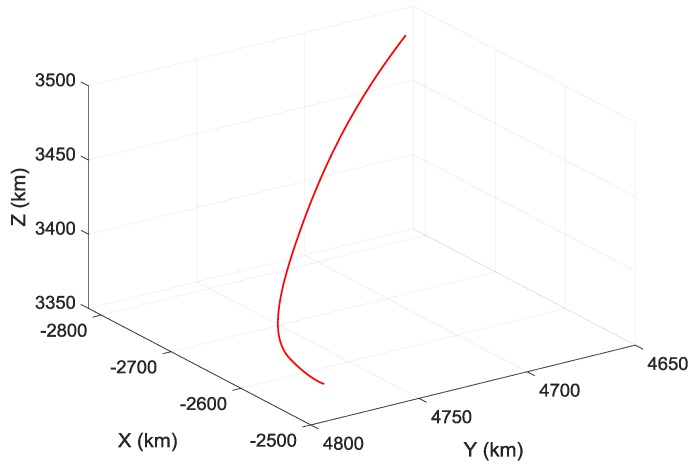
Standard flight path in geocentric coordinate.

**Figure 5:**
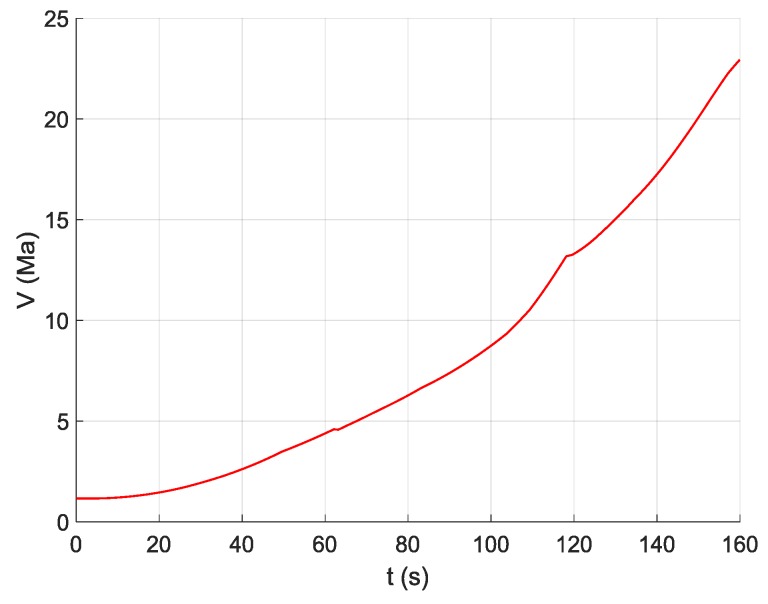
Velocity curve of aircraft in inertial coordinate.

This study aims to take high-speed and high-dynamic aircrafts as the research objects, such as ballistic missiles, hypersonic vehicles, and realize the error parameters estimation of SINS during the ascent phase flight of aircraft. Suppose that the aircraft employs a three-stage rocket to achieve flight, aircraft flight time of ascent phase is $T_{k}=160$ s, and SINS navigation cycle is 0.02 s. To generate signal excitation to SINS and realize the effective estimation of error parameters, the standard flight path of ascent phase of aircraft is designed in geocentric coordinate, as shown in Figure 4. Figure 5 shows the velocity curve of the aircraft in inertial coordinate.

### 5.2. Sample Generation and RBM-BPNN Structure Training

Training sample data should be collected to the train RBM-BPNN structure. The initial alignment error parameters of SINS and IMU instrument error parameters set in Section 5.1 are taken as 1 standard deviation σ, and 5000 groups of SINS error parameters are randomly generated in the form of normal distribution N(0,σ), which are taken as the training sample outputs of the neural network. We can obtain 5000 groups of navigation state parameters with navigation error, including position, velocity and attitude, by injecting 5000 groups of error parameters into the flight simulation software of the aircraft. The IMU error compensation model and SINS navigation calculation model of the software are consistent with the onboard computer of the aircraft to ensure consistency and accuracy of the model. The sum of position deviation (δx,δy,δz), the sum of velocity deviation $\left(\delta v_{x},\delta v_{y},\delta v_{z}\right)$ and the sum of attitude deviation (δφ,δψ,δγ) can be calculated based on SINS/GPS/CNS integrated navigation system measurement information and Equation (16)–(18). Thus, 5000 groups of navigation state parameter errors can be obtained, which are taken as the training sample inputs of the neural network.

**Figure 6:**
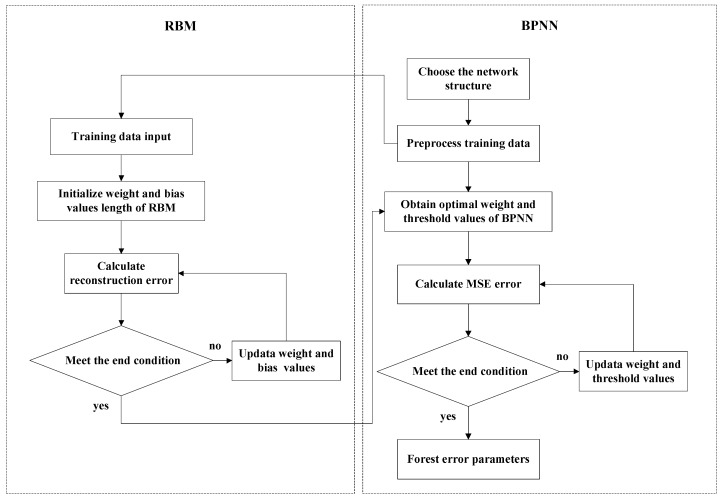
Training flow chart of RBM-BPNN.

According to the sample inputs and sample outputs and Equation (19), it can be seen that the number of input layer nodes is *n* = 9, and the number of the output layer nodes is *q* = 15. The number of hidden layer nodes *m* can be determined by using equation $m=\sqrt{n+q}+a$, where *a* is a constant in the interval [1,10], and *m* is chosen as 14 in this paper. The neural network activation function is chosen as the sigmoid function, RBM learning rate is η=0.0001, and BPNN learning rate is $\eta^{\prime }=0.01$. RBM and BPNN error training times are set to 1000. RBM-BPNN structure can be trained based on the above information, and the training flow chart of RBM-BPNN is shown in Figure 6.

### 5.3. Error Parameter Forecast Results by RBM-BPNN

**Figure 7:**
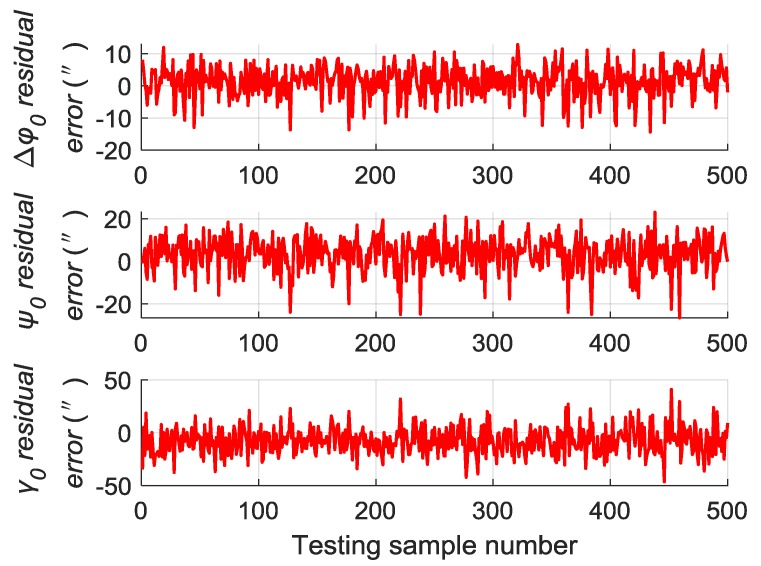
Simulation results of initial alignment residual error using RBM-BPNN method.

**Figure 8:**
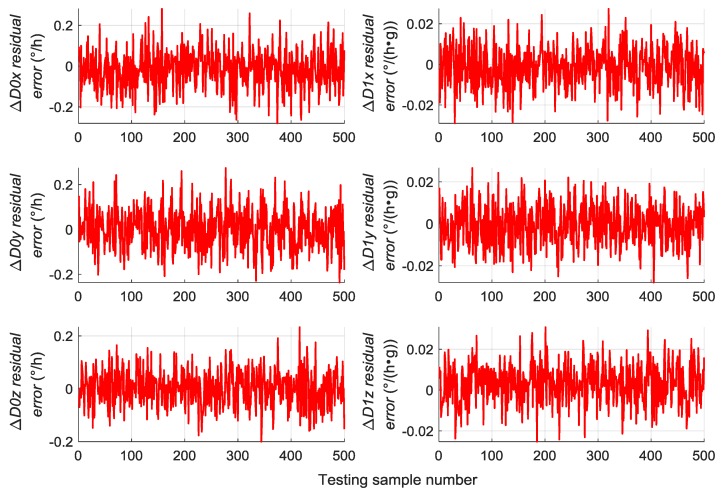
Simulation results of gyroscope residual error using RBM-BPNN method.

**Figure 9:**
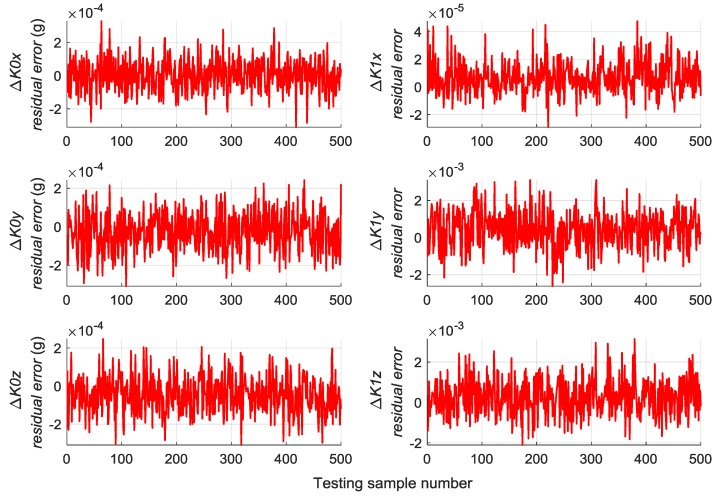
Simulation results of accelerometer residual error using RBM-BPNN method.

A total of 500 groups of data are randomly generated similar to Section 5.2 to verify the generalization capability of the trained RBM-BPNN structure, which are used as testing samples to evaluate the trained RBM-BPNN structure. According to simulation and calculation, the simulation results of initial alignment residual error using RBM-BPNN method is shown in Figure 7, the simulation results of gyroscope residual error using RBM-BPNN method is shown in Figure 8, and the simulation results of accelerometer residual error using RBM-BPNN method is shown in Figure 9. The residual error is the deviation between the actual value and the forecast value. Statistical results of error parameter forecast values using RBM-BPNN method are shown in Table 2.

As shown in Figure 7, Figure 8 and Figure 9 and Table 2, the RBM-BPNN method has high forecast accuracy for the initial alignment error parameters. The maximum value of initial pitch angle error and initial yaw angle error is not more than 30″, and the maximum value of initial roll angle error is not more than 50″. The MAE values of the initial alignment error are less than 20″, and the MAPE values are less than 30%. The RBM-BPNN method has poor forecast effect on gyroscope error parameters, and the MAPE values of gyroscope errors are more than 60%. For accelerometer error parameter, the RBM-BPNN method forecast effect is general, the MAPE values of accelerometer errors are generally more than 60%, but the forecast accuracy of accelerometer error parameter $\Delta K_{1x}$ is relatively high, and its MAPE value is 21.05%. This condition is because the axial acceleration of the body is the largest and has a good incentive effect on the estimation of the parameter $\Delta K_{1x}$. In general, the RBM-BPNN method has certain forecast effect for error parameters of SINS, especially for the initial alignment error parameters. However, the forecast accuracy of IMU instrument error parameters is poor, the navigation accuracy requirements of high dynamic and high-speed aircraft are difficult to meet, and the forecast accuracy of SINS error parameters should be improved.

### 5.4. RBM-BPNN Method Improvement and Simulation

As discussed in Section 5.3, the RBM-BPNN method has poor accuracy in forecasting SINS error parameters. The RBM-BPNN structure is improved to enhance the forecast effect of error parameters.

The inputs of RBM-BPNN structure are the sum of position deviation, the sum of velocity deviation and the sum of attitude deviation, which are obtained through the information fusion of SINS/GPS/CNS integrated navigation system. It should be pointed out that the inputs of neural network structure only utilize the navigation state parameter information. The gyroscope pulse and accelerometer pulse outputs of SINS are increased to the inputs of RBM-BPNN structure by utilizing the integrated navigation system information to realize the effective estimation of error parameters. This strategy is mainly adopted because the variation of SINS error parameters will generate the navigation state parameter deviation and change the output value of pulse signals. Therefore, the navigation state parameter deviation and pulse signal are used as the inputs of RBM-BPNN to obtain sufficient excitation information, which is beneficial in forecasting SINS error parameters.

The pulse signal should be processed before utilizing it as the input of RBM-BPNN, and the processing strategy of gyroscope pulse signal is shown in Equation (23):

(23) $$\left\{ \begin{array}{l} NB_{x1}=\sum_{t=0}^{T_{k}}Nb_{x}(t)\;Nb_{x}(t)<0\\ NB_{x2}=\sum_{t=0}^{T_{k}}Nb_{x}(t)\;Nb_{x}(t)\geq 0\\ NB_{y1}=\sum_{t=0}^{T_{k}}Nb_{y}(t)\;Nb_{y}(t)<0\\ NB_{y2}=\sum_{t=0}^{T_{k}}Nb_{y}(t)\;Nb_{y}(t)\geq 0\\ NB_{z1}=\sum_{t=0}^{T_{k}}Nb_{z}(t)\;Nb_{z}(t)<0\\ NB_{z2}=\sum_{t=0}^{T_{k}}Nb_{z}(t)\;Nb_{z}(t)\geq 0 \end{array}\right.$$

where $Nb_{x}(t)$, $Nb_{y}(t)$ and $Nb_{z}(t)$ denote the gyroscope pulse outputs at t moment; $NB_{i1}$ denotes the sum of gyroscope pulse $Nb_{i}(t)$ with its pulse value less than 0; $NB_{i2}$ denotes the sum of gyroscope pulse $Nb_{i}(t)$ with its pulse value greater than or equal to 0; i=x,y,z denote x, y and z axes, respectively. 

Similarly, the processing strategy of accelerometer pulse signal is shown in Equation (24):

(24) $$\left\{ \begin{array}{l} NA_{x1}=\sum_{t=0}^{T_{k}}Na_{x}(t)\;Na_{x}(t)<0\\ NA_{x2}=\sum_{t=0}^{T_{k}}Na_{x}(t)\;Na_{x}(t)\geq 0\\ NA_{y1}=\sum_{t=0}^{T_{k}}Na_{y}(t)\;Na_{y}(t)<0\\ NA_{y2}=\sum_{t=0}^{T_{k}}Na_{y}(t)\;Na_{y}(t)\geq 0\\ NA_{z1}=\sum_{t=0}^{T_{k}}Na_{z}(t)\;Na_{z}(t)<0\\ NA_{z2}=\sum_{t=0}^{T_{k}}Na_{z}(t)\;Na_{z}(t)\geq 0 \end{array}\right.$$

where $Na_{x}(t)$, $Na_{y}(t)$ and $Na_{z}(t)$ denote the accelerometer pulse outputs at t moment; $Na_{i1}$ denotes the sum of accelerometer pulse $Na_{i}(t)$ with its pulse value less than 0; $Na_{i2}$ denotes the sum of accelerometer pulse $Na_{i}(t)$ with its pulse value greater than or equal to 0.

It should be noted that the positive and negative values of pulse are distinguished when the pulse signal is processed by using Equation (23) and (24), this strategy can prevent the pulse summation calculation from offsetting the positive and negative pulses, so as to obtain considerable useful pulse information, which is conducive in the estimation of SINS error parameters. 

Therefore, the outputs of improved RBM-BPNN structure remain the same, and the inputs increase the pulse information in addition to the navigation state parameter deviations. The mapping relationship between the inputs and outputs of improved RBM-BPNN is adjusted as follows:

(25) $$\begin{array}{l} \left(\Delta \varphi_{0},\psi_{0},\gamma_{0},\Delta D_{0x},\Delta D_{0y},\Delta D_{0z},\Delta D_{1x},\Delta D_{1y},\Delta D_{1z},\Delta K_{0x},\Delta K_{0y},\Delta K_{0z},\Delta K_{1x},\Delta K_{1y},\Delta K_{1z}\right)\\ =f(\delta x,\delta y,\delta z,\delta v_{x},\delta v_{y},\delta v_{z},\delta \varphi ,\delta \psi ,\delta \gamma ,NB_{x1},NB_{x2},NB_{y1},NB_{y2},NB_{z1},NB_{z2},NA_{x1},NA_{x2},NA_{y1},NA_{y2},NA_{z1},NA_{z2}) \end{array}$$

This method is called the IRBM -BPNN method, which uses navigation state parameter deviation and pulse signal as inputs. 

**Figure 10:**
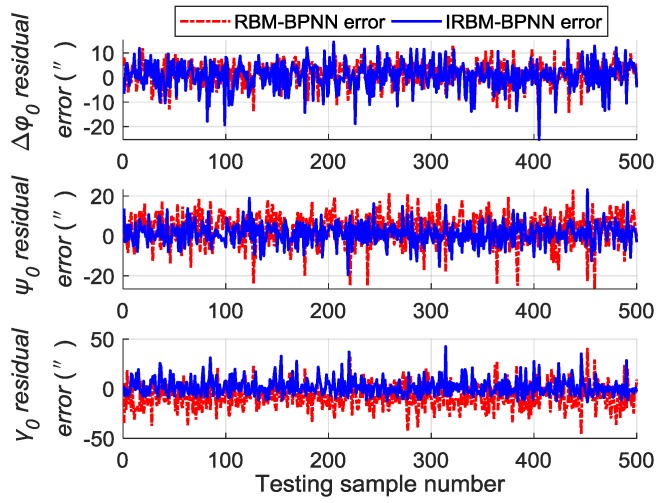
Contrast results of initial alignment residual error using RBM-BPNN and IRBM-BPNN method.

**Figure 11:**
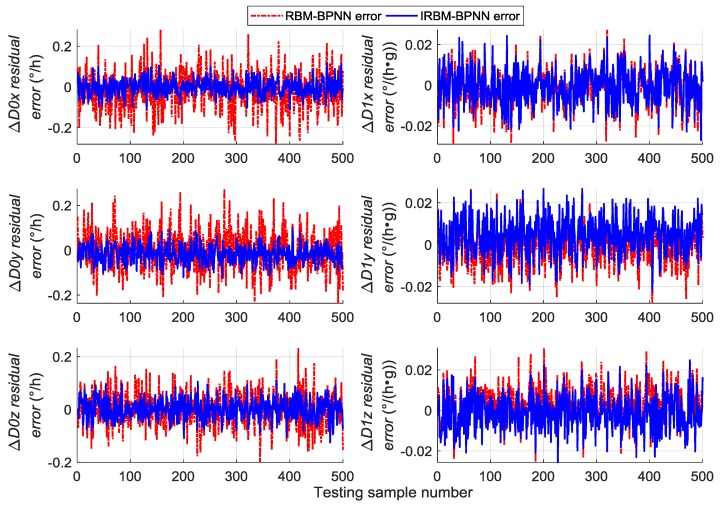
Contrast results of gyroscope residual error using RBM-BPNN and IRBM-BPNN method.

**Figure 12:**
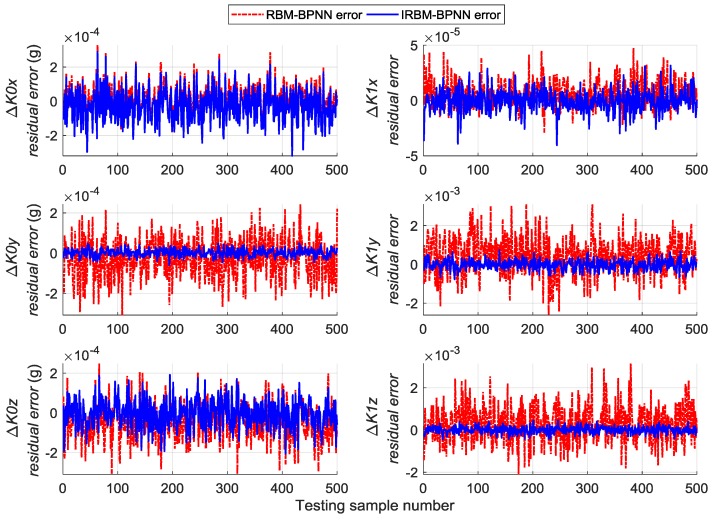
Contrast results of accelerometer residual error using RBM-BPNN and IRBM-BPNN method.

Next, the error parameters of the above 500 groups of test samples are forecasted by the IRBM-BPNN method to verify the feasibility of the proposed method. After simulation experiments, the residual errors of the error parameter forecast values using RBM-BPNN method and IRBM-BPNN method are shown in Figure 10, Figure 11 and Figure 12. Table 3 shows the statistical results of error parameter forecast values using IRBM-BPNN method.

As shown in Figure 10, Figure 11 and Figure 12, the IRBM-BPNN method has smaller residual error on the error parameter forecast values of SINS, and the forecast accuracy is obviously improved compared with the RBM-BPNN method. As can be seen from Table 3, the IRBM-BPNN method has high forecast effect on the initial alignment error parameter. The maximum value of the initial alignment errors is not more than 45″, the MAE values are less than 10″, and the MAPE values are less than 20%. The forecast accuracy of gyroscope error of the IRBM-BPNN method is better than that of RBM-BPNN method, and the MAPE values of gyroscope zero-order error parameters are less than 55% by using the IRBM-BPNN method. For the accelerometer error parameters, the IRBM-BPNN method shows high forecast level, and the MAPE values of parameters $\Delta K_{0y},\Delta K_{1y},\Delta K_{1z}$ are less than 40%. Especially, the MAPE value of parameter $\Delta K_{1x}$ is less than 20%. Therefore, it is feasible to improve the forecast accuracy of SINS error parameters by improving the RBM-BPNN structure and increasing the pulse signal as the network inputs. The IRBM-BPNN method has better forecast effect compared with the RBM-BPNN method.

### 5.5. Design of Simulation Comparison Experiments

**Figure 13:**
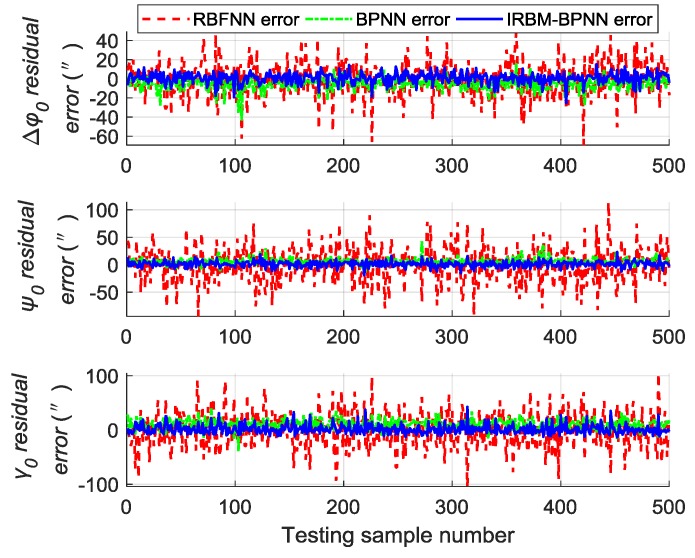
Contrast results of initial alignment residual error using three methods.

**Figure 14:**
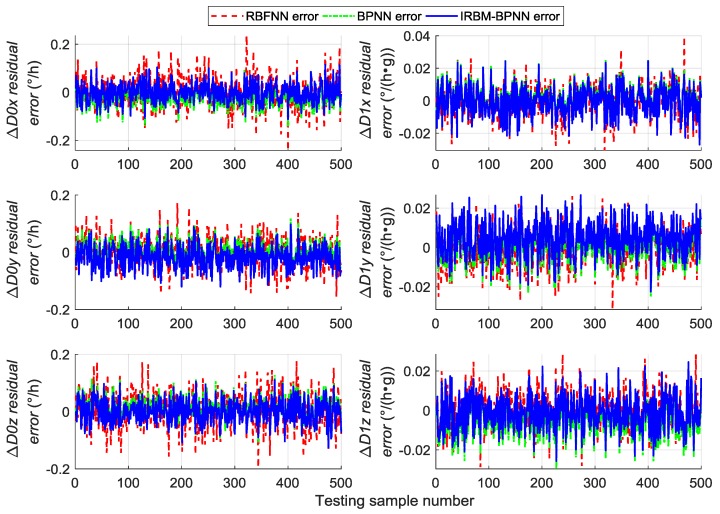
Contrast results of gyroscope residual error using three methods.

**Figure 15:**
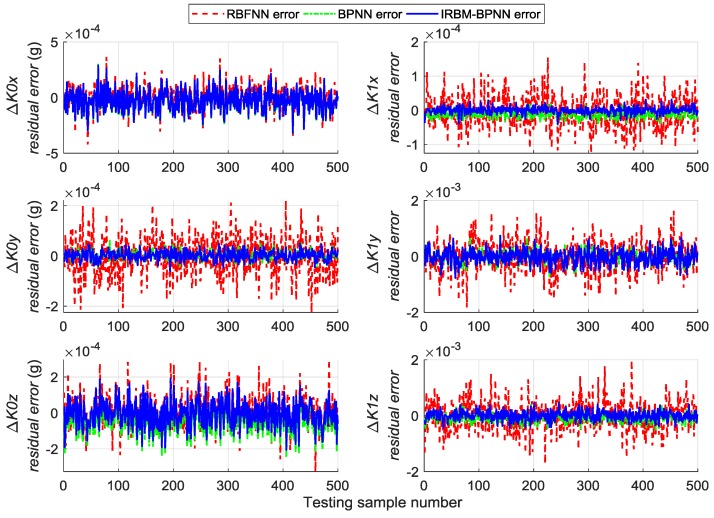
Contrast results of accelerometer residual error using three methods.

Comparative experiments are performed under the same simulation conditions, and the inputs and outputs of the training sample are consistent with Section 5.4 to further verify the effectiveness and feasibility of the IRBM-BPNN method in forecasting SINS error parameters. The RBFNN method and BPNN method are introduced as the comparative reference, and the radial base function of RBFNN is chosen as the Gaussian function. The statistical results of error parameter forecast values of testing samples by using the RBFNN method, BPNN method and IRBM-BPNN method are shown in Table 4. The residual error results using the three methods are shown in Figure 13, Figure 14 and Figure 15.

From Table 4 and Figure 13, Figure 14 and Figure 15, we can see that the three neural network methods have a good estimation effect on the initial alignment error, and the MAE values are less than 30″. This condition indicates that the artificial neural network method has certain advantages in forecasting SINS error parameters. For the forecast effect of IMU instrument error parameters, the BPNN method is better than the RBFNN method, whereas the IRBM-BPNN method is better than the BPNN method. The application of RBM to the initialization of BPNN can prevent the neural network from falling into local optimal, and the combination of RBM and BPNN methods can improve the forecast accuracy of SINS error parameters. At the same time, it is shown from Table 4 that the forecast effect of gyroscope first-order error parameters $\Delta D_{1x},\Delta D_{1y},\Delta D_{1z}$ and accelerometer zero-order error parameters $\Delta K_{0x},\Delta K_{0z}$ by the three neural network methods are not ideal. Their MAPE values are more than 60%, and research and improvement should be further conducted.

In summary, the IRBM-BPNN method has the highest forecast accuracy of SINS error parameters compared with the RBFNN method and BPNN method, which is feasible and effective in forecasting the initial alignment errors and IMU instrument errors of SINS for aircraft. 

Simulation experiments are performed again to validate the improved effect of SINS error forecast and aircraft navigation accuracy based on the IRBM-BPNN method. The initial alignment error parameters and the IMU instrument error parameters in Section 5.1 are set as the actual error parameters, and the actual error parameters are estimated under the same simulation conditions by the RBFNN method, BPNN method and IRBM-BPNN method. The forecast results of SINS error parameter by the RBFNN, BPNN and IRBM-BPNN methods through simulation are shown in Table 5.

From Table 5, we can find that RBFNN method has a good forecast effect on error parameters $\Delta \varphi_{0},\gamma_{0},\Delta D_{0x},\Delta D_{0y},\Delta D_{0z},\Delta D_{1y},\Delta D_{1z}\;\mathrm{and}\;\Delta K_{0x}$, in which their relative errors are less than 40%. The BPNN method has a good forecast effect on error parameters $\Delta \varphi_{0},\psi_{0},\gamma_{0},\Delta D_{0y},\Delta D_{0z},\Delta K_{0y},\Delta K_{0z},\Delta K_{1x},\Delta K_{1y}\;\mathrm{and}\;\Delta K_{1z}$, in which their relative errors are less than 30%. The IRBM-BPNN method has a good forecast effect on error parameters $\Delta \varphi_{0},\psi_{0},\gamma_{0},\Delta D_{0x},\Delta D_{0z},\Delta D_{1z},\Delta K_{0y},\Delta K_{0z},\Delta K_{1x},\Delta K_{1y}\;\mathrm{and}\;\Delta K_{1z}$, in which their relative errors are less than 30%. Obviously, the IRBM-BPNN method has the optimal forecast accuracy of SINS error parameters.

**Figure 16:**
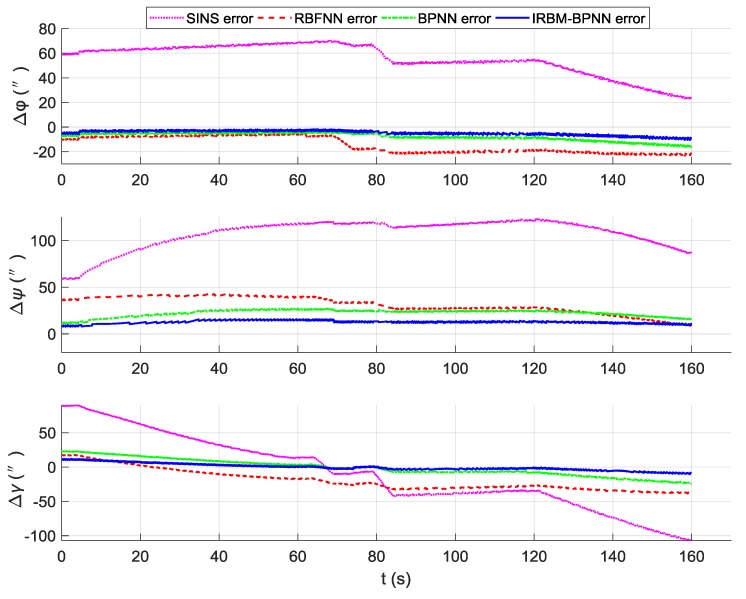
Contrast results of attitude error using three methods.

**Figure 17:**
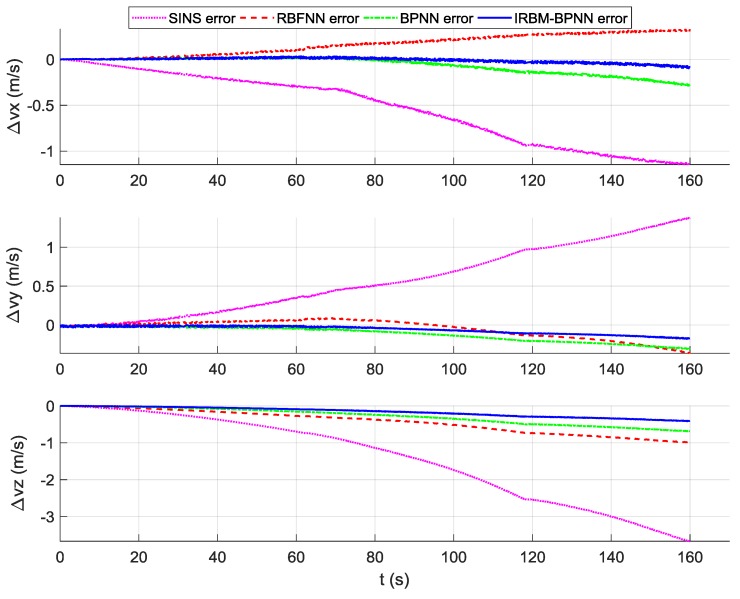
Contrast results of velocity error using three methods.

**Figure 18:**
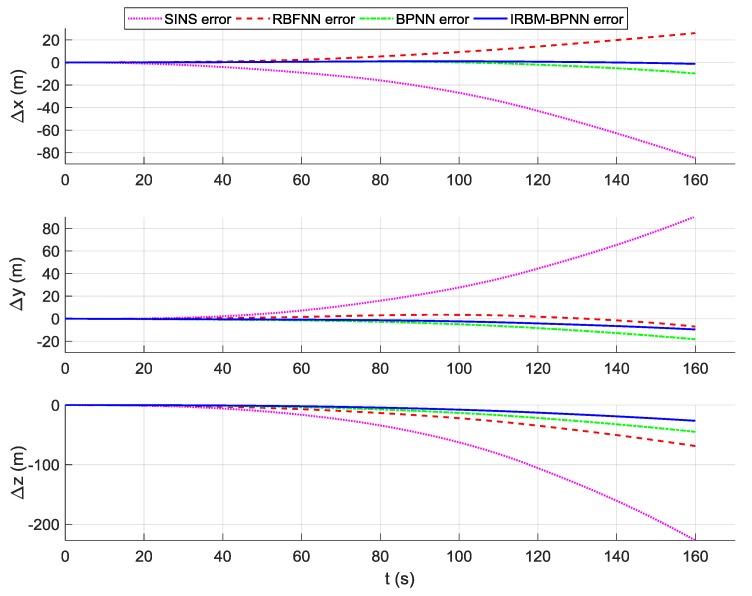
Contrast results of position error using three methods.

According to the overall scheme design shown in Figure 1, the IMU instrument error parameter forecast values of the artificial neural network method are fed back to the IMU error compensation model for compensation, and the initial alignment error parameter forecast values are fed back to the initial alignment error model for compensation. After error compensation and correction, the attitude error, velocity error and position error of the aircraft using the artificial neural network method can be obtained, as shown in Figure 16, Figure 17, and Figure 18. Table 6 shows the root mean square (RMS) error statistics of navigation parameters. 

As shown in Figure 16, Figure 17 and Figure 18, the attitude error, velocity error and position error are smaller than the pure SINS navigation error after the compensation and correction of the error parameters by the artificial neural network method. Therefore, the artificial neural network method can effectively improve the navigation accuracy of aircraft. As shown in Table 6, the RMS errors of attitude of the IRBM-BPNN method are less than 15″, which are smaller than the attitude errors of pure SINS navigation and the smallest among the attitude errors of the RBFNN method and BPNN method. The RMS errors of position and velocity of the IRBM-BPNN method are less than 15 m and 0.25 m/s, respectively. Obviously, the navigation errors of the IRBM-BPNN method are the smallest compared with the RBFNN method and BPNN method. Therefore, the IRBM-BPNN method has the optimal navigation accuracy.

## 6. Conclusions

In this paper, a hybrid IRBM-BPNN approach that combines RBM and BPNN is proposed to estimate the IMU instrument error parameters and initial alignment error parameters of SINS for aircraft. From the view of machine learning, the network initialization of BPNN is realized by RBM. The RBM-BPNN forecast model takes the navigation deviations as the inputs and SINS error parameters as the outputs based on the measurement information of SINS/GPS/CNS integrated navigation system. The RBM-BPNN structure is improved to enhance the forecast accuracy of SINS error parameters, and the error parameters are forecasted and compensated by the IRBM-BPNN method. The simulation results show that the forecast performance of the neural network can be effectively improved by combining RBM and BPNN methods and improving the RBM-BPNN network structure. The IRBM-BPNN method has the highest forecast accuracy for SINS error parameters and can effectively improve the navigation accuracy of aircraft compared with the RBFNN method and BPNN method. 

This paper provides new insight into the estimation of initial alignment error parameters and IMU instrument error parameters of SINS by introducing the unsupervised machine learning mechanism. This study mainly conducts a detailed analysis and demonstrates for SINS error parameter estimation of aircraft at the theoretical and simulation levels, and the reliability and stability of the proposed method should be further verified through flight testing in the future. On the whole, with the rapid development of artificial intelligence and deep learning technology, the advantages of artificial neural network will increase, and the estimation of SINS error parameters for aircraft based on artificial neural network and deep learning will become a hot research topic in the future. 

## Figures and Tables

**Table 1:** Instrument error parameters of IMU.

Senor	Error Parameters	Error Value	Units
Gyroscope	Zero-order error $\Delta D_{0x}$	0.1	°/h
	Zero-order error $\Delta D_{0y}$	0.1	°/h
	Zero-order error $\Delta D_{0z}$	0.1	°/h
	First-order error $\Delta D_{1x}$	0.01	°/(h⋅g)
	First-order error $\Delta D_{1y}$	0.01	°/(h⋅g)
	First-order error $\Delta D_{1z}$	0.01	°/(h⋅g)
	White noise $\varepsilon_{g}$	0.01	°/h
Accelerometer	Zero-order error $\Delta K_{0x}$	1.0 × 10^−4^	g
	Zero-order error $\Delta K_{0y}$	1.0 × 10^−4^	g
	Zero-order error $\Delta K_{0z}$	1.0 × 10^−4^	g
	First-order error $\Delta K_{1x}$	1.0 × 10^−4^	-
	First-order error $\Delta K_{1y}$	1.0 × 10^−^^3^	-
	First-order error $\Delta K_{1z}$	1.0 × 10^−^^3^	-
	White noise $\varepsilon_{a}$	1.0 × 10^−^^5^	g

**Table 2:** Statistical results of error parameter forecast values using RBM-BPNN method.

Error Parameter	Maximum Value	Minimum Value	MAE	MAPE/(%)
$\Delta \varphi_{0}$/(″)	12.8593	−14.3543	4.0847	17.69
$\psi_{0}$/(″)	23.1374	−26.5427	7.1069	27.72
$\gamma_{0}$/(″)	41.0735	−46.3723	12.0742	28.41
$\Delta D_{0x}$/(°/h)	2.8065 × 10^−^^1^	−2.8068 × 10^−^^1^	7.5766 × 10^−^^2^	85.63
$\Delta D_{0y}$/(°/h)	2.7374 × 10^−^^1^	−2.3656 × 10^−^^1^	7.3499 × 10^−^^2^	78.21
$\Delta D_{0z}$/(°/h)	2.3289 × 10^−^^1^	−2.0062 × 10^−^^1^	5.3618 × 10^−^^2^	62.39
$\Delta D_{1x}$/(°/(h⋅g))	2.7299 × 10^−^^2^	−2.9003 × 10^−^^2^	7.9876 × 10^−^^3^	92.51
$\Delta D_{1y}$/(°/(h⋅g))	2.6547 × 10^−^^2^	−2.7958 × 10^−^^2^	7.8484 × 10^−^^3^	87.86
$\Delta D_{1z}$/(°/(h⋅g))	3.0782 × 10^−^^2^	−2.5271 × 10^−^^2^	8.1292 × 10^−^^3^	81.94
$\Delta K_{0x}$/(g)	3.2797 × 10^−^^4^	−3.1048 × 10^−^^4^	7.5387 × 10^−^^5^	92.22
$\Delta K_{0y}$/(g)	2.4197 × 10^−^^4^	−3.0791 × 10^−^^4^	8.1093 × 10^−^^5^	81.02
$\Delta K_{0z}$/(g)	2.4728 × 10^−^^4^	−3.0946 × 10^−^^4^	8.6272 × 10^−^^5^	77.54
$\Delta K_{1x}$	4.7426 × 10^−^^5^	−2.9022 × 10^−^^5^	1.0119 × 10^−^^5^	21.05
$\Delta K_{1y}$	3.0925 × 10^−^^3^	−2.6130 × 10^−^^3^	8.8878 × 10^−^^4^	83.01
$\Delta K_{1z}$	3.1416 × 10^−^^3^	−2.0996 × 10^−^^3^	7.4163 × 10^−^^4^	79.66

**Table 3:** Statistical results of error parameter forecast values using IRBM-BPNN method.

Error Parameter	Maximum Value	Minimum Value	MAE	MAPE/(%)
$\Delta \varphi_{0}$/(″)	15.1057	−25.2937	4.2752	17.32
$\psi_{0}$/(″)	23.1410	−19.3652	4.2384	17.57
$\gamma_{0}$/(″)	42.4832	−13.4244	6.3805	17.24
$\Delta D_{0x}$/(°/h)	1.1449 × 10^−^^1^	−1.1188 × 10^−^^1^	3.2479 × 10^−^^2^	52.06
$\Delta D_{0y}$/(°/h)	9.7487 × 10^−^^2^	−1.2024 × 10^−^^1^	3.4799 × 10^−^^2^	53.14
$\Delta D_{0z}$/(°/h)	1.1065 × 10^−^^1^	−1.2577 × 10^−^^1^	3.2075 × 10^−^^2^	47.47
$\Delta D_{1x}$/(°/(h⋅g))	2.4441 × 10^−^^2^	−2.7048 × 10^−^^2^	7.2427 × 10^−^^3^	79.74
$\Delta D_{1y}$/(°/(h⋅g))	2.6639 × 10^−^^2^	−2.2403 × 10^−^^2^	7.9801 × 10^−^^3^	76.89
$\Delta D_{1z}$/(°/(h⋅g))	2.4528 × 10^−^^2^	−2.5661 × 10^−^^2^	6.9553 × 10^−^^3^	72.46
$\Delta K_{0x}$/(g)	2.8906 × 10^−^^4^	−3.1930 × 10^−^^4^	7.6031 × 10^−^^5^	82.18
$\Delta K_{0y}$/(g)	4.8948 × 10^−^^5^	−6.0603 × 10^−^^5^	1.4845 × 10^−^^5^	32.22
$\Delta K_{0z}$/(g)	1.9109 × 10^−^^4^	−2.0527 × 10^−^^4^	5.7869 × 10^−^^5^	67.25
$\Delta K_{1x}$	3.1575 × 10^−^^5^	−4.0429 × 10^−^^5^	7.2550 × 10^−^^6^	17.17
$\Delta K_{1y}$	7.3813 × 10^−^^4^	−7.6925 × 10^−^^4^	2.0235 × 10^−^^4^	38.87
$\Delta K_{1z}$	4.6047 × 10^−^^4^	−4.4187 × 10^−^^4^	1.2891 × 10^−^^4^	30.32

**Table 4:** Statistical results of error parameter forecast values of testing samples.

Error parameter	RBFNN		BPNN		IRBM-BPNN	
	MAE	MAPE/(%)	MAE	MAPE/(%)	MAE	MAPE/(%)
$\Delta \varphi_{0}$/(″)	14.4716	39.47	6.7169	23.55	4.2752	17.32
$\psi_{0}$/(″)	25.3949	55.02	6.9855	25.24	4.2384	17.57
$\gamma_{0}$/(″)	26.1093	44.47	12.0992	30.68	6.3805	17.24
$\Delta D_{0x}$/(°/h)	5.1322 × 10^−2^	63.57	3.7878 × 10^−2^	56.65	3.2479 × 10^−2^	52.06
$\Delta D_{0y}$/(°/h)	4.4252 × 10^−^^2^	58.66	3.2558 × 10^−2^	51.09	3.4799 × 10^−2^	53.14
$\Delta D_{0z}$/(°/h)	4.8451 × 10^−2^	58.59	3.3190 × 10^−2^	47.74	3.2075 × 10^−2^	47.47
$\Delta D_{1x}$/(°/(h⋅g))	8.3080 × 10^−3^	80.47	7.3119 × 10^−3^	79.45	7.2427 × 10^−3^	79.74
$\Delta D_{1y}$/(°/(h⋅g))	8.2976 × 10^−3^	79.67	7.1242 × 10^−3^	76.23	7.9801 × 10^−3^	76.89
$\Delta D_{1z}$/(°/(h⋅g))	8.0855 × 10^−3^	77.00	8.0056 × 10^−3^	76.19	6.9553 × 10^−3^	72.46
$\Delta K_{0x}$/(g)	8.6713 × 10^−5^	82.33	7.7971 × 10^−5^	80.14	7.6031 × 10^−5^	82.18
$\Delta K_{0y}$/(g)	6.0672 × 10^−5^	70.41	1.5862 × 10^−5^	34.22	1.4845 × 10^−5^	32.22
$\Delta K_{0z}$/(g)	7.6700 × 10^−5^	78.39	6.7325 × 10^−5^	70.40	5.7869 × 10^−5^	67.25
$\Delta K_{1x}$	3.7834 × 10^−5^	52.52	1.4592 × 10^−5^	32.65	7.2550 × 10^−^^6^	17.17
$\Delta K_{1y}$	4.4197 × 10^−4^	59.16	2.0157 × 10^−4^	38.75	2.0235 × 10^−4^	38.87
$\Delta K_{1z}$	4.4192 × 10^−4^	60.16	1.5053 × 10^−4^	33.97	1.2891 × 10^−4^	30.32

**Table 5:** Forecast results of error parameter settings by three methods.

Error Parameter	Actual Value	RBFNN		BPNN		IRBM-BPNN	
		Estimated Value	Relative Error /(%)	Estimated Value	Relative Error /(%)	Estimated Value	Relative Error /(%)
$\Delta \varphi_{0}$/(″)	60	69.250841	15.42	66.372812	10.62	64.152852	6.92
$\psi_{0}$/(″)	60	22.492555	62.51	47.194597	21.34	50.519926	15.80
$\gamma_{0}$/(″)	90	71.941863	20.06	66.508608	26.10	78.082916	13.24
$\Delta D_{0x}$/(°/h)	0.1	0.104901	4.90	0.145416	45.42	0.115932	15.93
$\Delta D_{0y}$/(°/h)	0.1	0.074452	25.55	0.120273	20.27	0.136983	36.98
$\Delta D_{0z}$/(°/h)	0.1	0.076391	23.61	0.077471	22.53	0.085497	14.50
$\Delta D_{1x}$/(°/(h⋅g))	0.01	−0.001552	100.00	0.003833	61.67	0.004844	51.56
$\Delta D_{1y}$/(°/(h⋅g))	0.01	0.013379	33.79	0.002648	73.52	0.001272	87.28
$\Delta D_{1z}$/(°/(h⋅g))	0.01	0.002397	76.03	0.013044	30.44	0.012514	25.13
$\Delta K_{0x}$/(g)	1.0 × 10^−^^4^	6.4540 × 10^−^^5^	35.46	1.2107 × 10^−^^5^	87.89	1.4009 × 10^−^^5^	85.99
$\Delta K_{0y}$/(g)	1.0 × 10^−^^4^	5.1649 × 10^−^^5^	48.35	7.0472 × 10^−^^5^	29.53	1.2214 × 10^−^^4^	22.14
$\Delta K_{0z}$/(g)	1.0 × 10^−^^4^	−7.7172 × 10^−^^5^	100.00	1.0760 × 10^−^^4^	7.60	7.1746 × 10^−^^5^	28.25
$\Delta K_{1x}$	1.0 × 10^−^^4^	1.3814 × 10^−^^5^	86.19	1.1293 × 10^−^^4^	12.93	9.5368 × 10^−^^5^	4.63
$\Delta K_{1y}$	1.0 × 10^−^^3^	1.9599 × 10^−^^4^	80.40	7.0701 × 10^−^^4^	29.30	8.4009 × 10^−^^4^	15.99
$\Delta K_{1z}$	1.0 × 10^−^^3^	1.5141 × 10^−^^3^	51.41	9.0074 × 10^−^^4^	9.93	8.2878 × 10^−^^4^	17.12

**Table 6:** RMS error statistics of navigation parameters.

Error Type	Navigation Parameter	Pure SINS Error	RBFNN Error	BPNN Error	IRBM-BPNN Error
Attitude error/(″)	φ	56.836476	15.927349	8.308803	5.122294
	ψ	108.665519	32.575822	22.702491	12.773308
	γ	53.972813	24.864880	12.464985	5.130052
Position error/(m)	x	35.629810	11.301681	2.967975	0.636830
	y	37.127953	2.393230	7.208955	3.692268
	z	90.433416	28.697697	18.219801	10.700961
Velocity error/(m/s)	$v_{x}$	0.649370	0.195083	0.108240	0.028812
	$v_{y}$	0.709428	0.124732	0.146494	0.078337
	$v_{z}$	1.824331	0.527373	0.354347	0.208763
